# A metabolomics-based analysis of the metabolic pathways associated with the regulation of branched-chain amino acids in rats fed a high-fructose diet

**DOI:** 10.1530/EC-23-0079

**Published:** 2023-09-08

**Authors:** Yang Yu, Hairong Hao, Linghui Kong, Jie Zhang, Feng Bai, Fei Guo, Pan Wei, Rui Chen, Wen Hu

**Affiliations:** 1 Department of Endocrinology and Metabolism, Huai’an Hospital Affiliated to Xuzhou Medical University and Huai’an Second People’s Hospital, Huai’an, Jiangsu, China

**Keywords:** fructose, skeletal muscle, branched-chain amino acids, metabolic disorders, metabonomics

## Abstract

Previous studies have shown that the elevated levels of circulating branched-chain amino acids (BCAAs) are associated with the development of insulin resistance and its complications, including obesity, type 2 diabetes, cardiovascular disease and some cancers. However, animal models that can mimic the metabolic state of chronically elevated BCAAs in humans are rare. Therefore, the aim of this study was to establish the above animal model and analyse the metabolic changes associated with high BCAA levels. Sixteen 8-week-old Sprague–Dawley (SD) rats were randomly divided into two groups and given either a high fructose diet or a normal diet. BCAA levels as well as blood glucose and lipid levels were measured at different time points of feeding. The mRNA expression levels of two key enzymes of BCAA catabolism, ACAD (acyl-CoA dehydrogenase) and BCKDH (branched-chain α-keto acid dehydrogenase), were measured by qPCR, and the protein expression levels of these two enzymes were analysed by immunohistochemistry. Finally, the metabolite expression differences between the two groups were analysed by Q300 metabolomics technology. Our study confirms that defects in the catabolic pathways of BCAAs lead to increased levels of circulating BCAAs, resulting in disorders of glucose and lipid metabolism characterized by insulin resistance by affecting metabolic pathways associated with amino acids and bile acids.

## Introduction

Branched-chain amino acids (BCAAs) are neutral amino acids containing branched aliphatic hydrocarbon chains on the α-carbon, including l-leucine, l-isoleucine and l-valine, which are essential amino acids that cannot be synthesized normally in the body and must be obtained through exogenous pathways (1) and which act on metabolic signalling pathways in direct and indirect ways. BCAA is mainly metabolized in skeletal muscle, accounting for approximately 35% of the essential amino acids of skeletal muscle proteins, and has a close relationship with skeletal muscle synthesis (2). However, excessive amino acid uptake or inborn errors in genes encoding catalytic enzymes in the catabolic pathway of BCAAs leads to the accumulation of BCAAs and their metabolites (3), which will produce pathological changes ranging from nerve to myocardium (4). In 2009, Newgard *et al.* (5) found that in obese individuals, elevated BCAA and its metabolites were significantly associated with insulin resistance. Since then, an increasing number of findings suggest (6, 7, 8, 9, 10) that elevated BCAA levels are associated with obesity, type 2 diabetes and cardiovascular disease (CVD). Our previous cross-sectional study of 1302 retired workers also confirmed that BCAA levels were independently and positively associated with dyslipidaemia (11), oxidative stress (12) and high cardiovascular risk (13) in Chinese elderly people. It is necessary to investigate the mechanism by which BCAAs affect these metabolic diseases.

The key point is to establish an animal model with steadily elevated BCAAs. For the animal experiments, a few studies used PP2Cm^−/−^ mice for the construction of animal models of hyperbranched-chain amino academia (14). PP2Cm, a mitochondrial-targeted phosphatase, specifically binds the BCKD complex. PP2Cm^−/−^ mice have significantly increased plasma BCAA levels, which is evidence of moderate BCAA catabolic abnormalities. PP2Cm-deficient mice exhibit defective BCAA catabolism and a metabolic phenotype similar to that of intermittent or intermediate human maple syrup urine disease, a genetic disorder caused by defective BCKD activity. Furthermore, when fed a high-protein diet, PP2Cm^−/−^ mice showed the induction of oxidative stress and elevated neonatal mortality. However, such knockout mice can only reflect the acute elevated changes in BCAA levels without obesity, dyslipidaemia, abnormal glucose tolerance, insulin resistance (15) and so on. They cannot fully mimic the pathological state of chronically elevated BCAA levels associated with metabolic diseases. They also cannot possess the clinical features of chronic metabolic diseases such as disorders of glucose and lipid metabolism. These animals are scarce and expensive. It is more difficult to make an animal model with steadily elevated branched-chain amino acids accompanied by metabolic disorders.

However, a study (16) revealed that rats fed a standard diet containing either starch or fructose had elevated blood BCAA levels and abnormal glucose metabolism with no changes in the liver or the adipose tissue (AT), but instead an impaired capacity of the skeletal muscle to catabolize BCAAs. It was speculated that rats might be a suitable animal model. Therefore, the aim of this study was to investigate the metabolic changes under high fructose and to initially explore the relationship between elevated BCAAs and other metabolic changes.

## Materials and methods

### Establishment of a rat model of hyperbranched-chain amino acidaemia induced by high fructose

Sixteen 8-week-old SPF-rated healthy male Sprague–Dawley (SD) rats, weighing approximately 300 g (Xuzhou Medical University Experimental Animal Center, Xuzhou, China), were housed in steel cages with a constant temperature of 18–22°C and 50–60% relative humidity in a thermostatic incubator. Food and water were supplied ad libitum and the light/darkness cycle was 12 h. The rats were randomly divided into two groups: the normal diet group (SD group) and the high fructose group (HFTD group, FBSH Biotechnology Co., Ltd., Shanghai, China), with eight rats in each group. The high-fructose diet in the HFTD group contained 60% fructose, while the control group was given the standard diet and fed continuously for 8 weeks. The specific dietary composition table is in Supplementary Table 1 (see section on supplementary materials given at the end of this article). After 8 weeks, serum BCAAs were detected to confirm the model establishment. All experiments on animals were approved by the animal ethics agency and the use committee of Xuzhou Medical University.

#### Body weight and serum biochemical indices of rats

The body weight of rats in the SD and HFTD groups was measured once a week for 8 weeks. Blood was taken from the tip of the tail after 12 h of fasting, and fasting blood was measured by the Freestyle Optium blood glucose and ketone body monitoring system (Abbott Diabetes Care Ltd., Oxford, UK) with an additional random blood glucose measurement. Fasting insulin was measured using a rat insulin ELISA kit (Beyotime, Shanghai, China) according to the instructions. Lipid quadruplicates and liver function were measured using a fully automated biochemical analyser (Shenzhen Radu Life Sciences, Shenzhen, China).

#### Detection of plasma BCAA levels in rats

The level of BCAAs in mouse plasma was measured by ELISA, and the specific steps and detection methods were performed according to the instructions of the BCAA level determination kit (Abcam). In short, 10 μL of the 10 mM leucine standard were diluted with 90 μL dH_2_O to generate 1 mM of leucine standard. About 0, 2, 4, 6, 8 and 10 μL of the diluted standard were added into a 96-well plate to generate 0, 2, 4, 6, 8 and 10 nmol/well standard. After finishing the standard curve preparation, 50 μL of the reaction mix were added to each well containing the leucine standard and test samples and mixed well. The reaction was incubated for 30 min at room temperature and was protected from light. Finally, OD was measured at 450 nm in a microplate reader.

#### Immunohistochemistry

Antigen retrieval was performed using Er2 buffer (Leica), and sections were incubated with acyl-CoA dehydrogenase (ACAD) (1:500, Servicebio) and BCKDH (1: 500, Servicebio) primary antibodies for 24 h at 4°C. Following incubation with anti-rat-HRP or anti-rabbit-HRP secondary antibodies, the sections were stained with DAB chromogenic substrate. Counterstaining was performed using haematoxylin. Images were obtained using an Eclipse Ti-S fluorescence microscope (Nikon).

#### Quantitative real-time polymerase chain reaction

TRIzol reagent (Roche) was used to isolate total RNA from tissues or cells, and a reverse transcription kit (Takara Bio) was used to synthesize cDNA. RT-PCR was conducted using Takara Bio’s SYBR Premix Ex Taq II reagent with the Bio-Rad RT-PCR system. Gene expression was quantified using the standard housekeeping gene as an internal loading control. Amplification specificity was confirmed by melting curves; fluorescence was determined at 60°C. The PCR conditions were as follows: predenaturation at 95°C for 30 s, followed by 40 cycles of 5 s at 95°C and 31 s at 60°C. qRT-PCRs were performed in a total volume of 20 µL. The sequence of gene primers is shown in Supplementary Table 2. All experiments were performed in triplicate. Relative quantitative expression levels were calculated using the 2^−∆∆^^Ct^ method.

#### Collection of serum samples and metabolomic analysis by Q300 microarray

At the end of the experiment, blood was collected from the hearts of 16 rats and centrifuged at 3500 ***g*** for 10 min after standing for 30 min at room temperature, and the supernatant was collected and stored at −80℃. Before analysis, the serum was thawed at 4℃ and vortexed for 30 s. About 100 μL of the sample were extracted by adding 300 μL of acetonitrile and 10 μL of μmol/L internal standard and then centrifuged at 4℃ at 4000 ***g*** for 20 min. About 330 μL of supernatant were taken, blown dry with nitrogen and then redissolved with 100 μL of ultrapure water at 4℃ and spun at 4000 ***g*** for 10 min; finally, 80 μL of supernatant were used for the Q300 metabolic chip full targeting assay (Shanghai Metropolis Biotechnology Co., Ltd.).

#### Statistical analysis

Statistical analysis was performed using IBM SPSS Statistics Version 19.0 (IBM Corp). The results are expressed as the mean ± s.d. Student’s *t*-test was used to compare data between the two groups. Variance analysis was performed to compare multiple groups. *P* < 0.05 was considered statistically significant.

### Results

#### Weight gain, elevated plasma levels of BCAAs, disorders of glucolipid metabolism and abnormal liver function in rats fed a high-fructose diet

**Figure 1:**
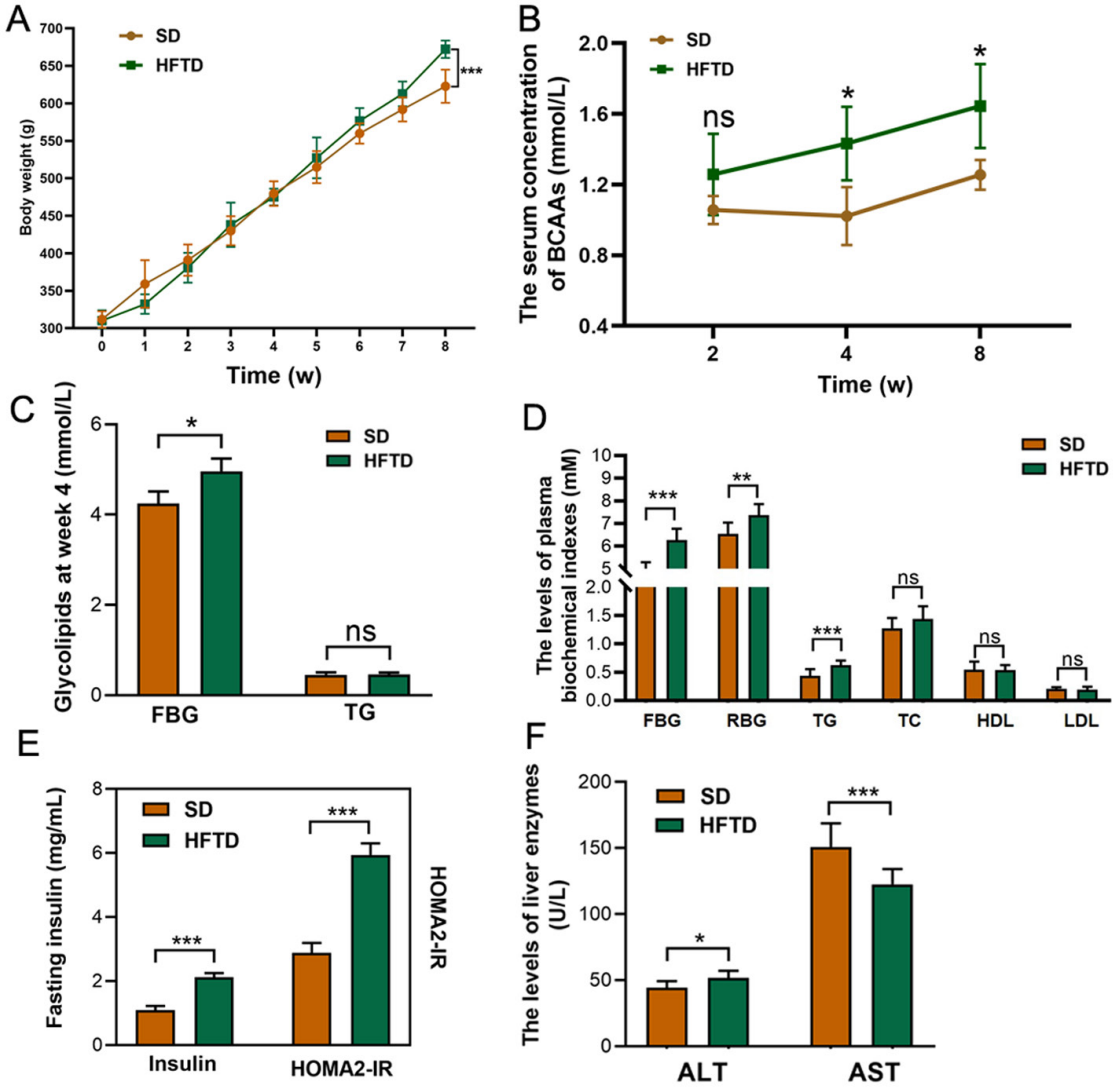
Increased body weight, plasma levels of BCAAs and abnormal serum biochemical parameters in rats on a high fructose diet. (A) Body weight graphs of control (males, *n* = 8) and high-fructose diet rats (males, *n* = 8) from 0 to 8 weeks. (B) Values of plasma BCAA concentrations measured at the second, fourth and eighth weeks of feeding in rats (males, *n* = 8), respectively. (C) Blood glucose and triglyceride were measured at week 4. (D–F) Serum biochemical parameters were summarized at week 8 from control (male, *n* = 8) to high-fructose diet group rats (male, *n* = 8). Data are presented as mean ± s.d. (*n* = 8). ^*^represents significance at *P* < 0.05,^**^ represents significance at *P* < 0.01, ^***^represents significance at *P* < 0.001, Student’s *t*-test.

After high-fructose diet feeding, the body weight of the rats was monitored for 8 weeks, and it was found that the body weight of the rats in the HFTD group was significantly higher than that of the control rats at the seventh and eighth weeks of feeding, and especially higher at the eighth week (*P* < 0.01, 650 and 600 g), but there was no significant difference between the two groups during the first 6 weeks (Fig. 1A). The levels of plasma BCAAs in the HFTD group gradually increased from the fourth week to the eighth week and were higher than those in the control group, which indicated that the high fructose-induced hyperbranched-chain amino acidaemia rat models were successfully established.

Next, the biochemical indices of the rats were further analysed (Fig. 1C, D, E and F). At the fourth week, the fasting blood glucose of the rats in the HFTD group was higher than that of the control rats (*P* = 0.047), but the TG levels of the two groups were not significantly different (*P* > 0.05) (Fig. 1C). By the end of the experiment, serum TG levels were elevated in the high-fructose-fed rats compared to the control group (*P* < 0.001), and total cholesterol (TC), high-density lipoprotein cholesterol (HDL-C) and low density lipoprotein cholesterol (LDL-C) levels were not significantly elevated. In addition, compared with the control group, fasting glucose, random glucose (RBG) and fasting insulin were significantly higher in high fructose-fed rats, accompanied by an elevated insulin resistance index (HOMA-IR) (Fig. 1D and E), which was statistically significant. Additionally, in terms of liver function, compared to control rats, rats in the HFTD group had increased serum alanine aminotransferase (ALT) levels and decreased aspartate aminotransferase (AST) levels, both of which were statistically significant (Fig. 1F).

#### Decreased expression levels of muscle BCAA catabolic enzymes (ACAD, BCKDH) in rats fed a high fructose diet

**Figure 2:**
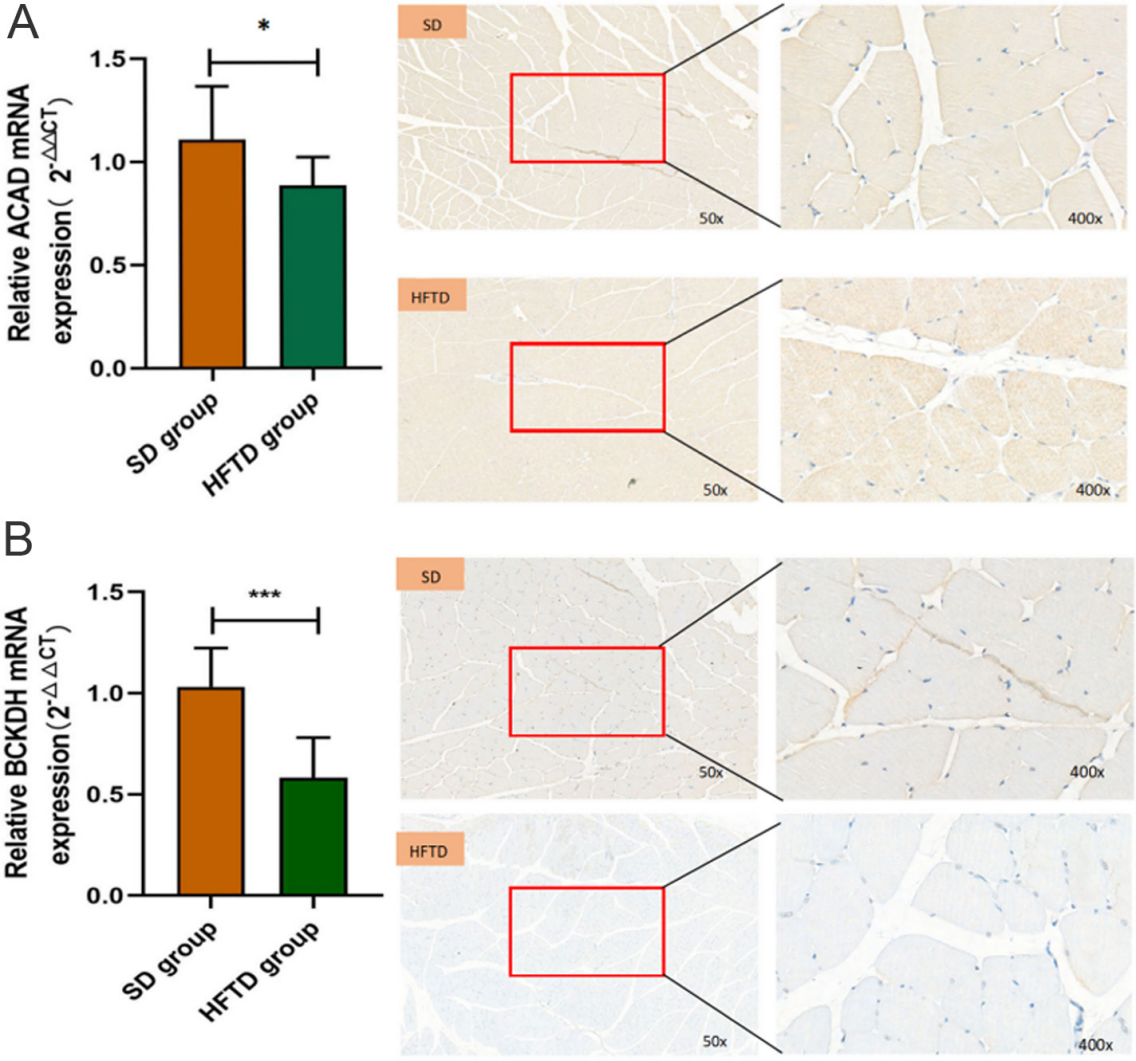
Defective skeletal muscle BCAA metabolism in rats fed a high fructose diet. (A, B) qPCR and immunohistochemistry analysis of two key enzymes in skeletal muscle catabolism of BCAA at week 8 of rat feeding. SD group (*n* = 8), HFTD group (*n* = 8). (A) *P* = 0.05 HFTD group vs SD group (ACAD), (B) *P* = 0.0004 HFTD group vs SD group (BCKDH).

The catabolism of BCAAs occurs mainly in muscle tissue. Therefore, the expression of BCAAs-metabolizing enzymes in rat skeletal hindlimb muscles was investigated. Branched-chain α-keto acid dehydrogenase (BCKDH) and ACAD are the two rate-limiting enzymes of BCAA catabolism. The mRNA expression levels of muscle BCAA catabolic enzymes (ACAD and BCKDH) were significantly lower in the HFTD rats than in control rats. Additionally, the BCKDH protein expression level was also significantly reduced in the HFTD group rats, but the ACAD protein expression level was not significantly different (Fig. 2A and B).

### Metabolomic analysis of rat serum Q300

#### Metabolic profile analysis

##### Metabolite classification overview

**Figure 3:**
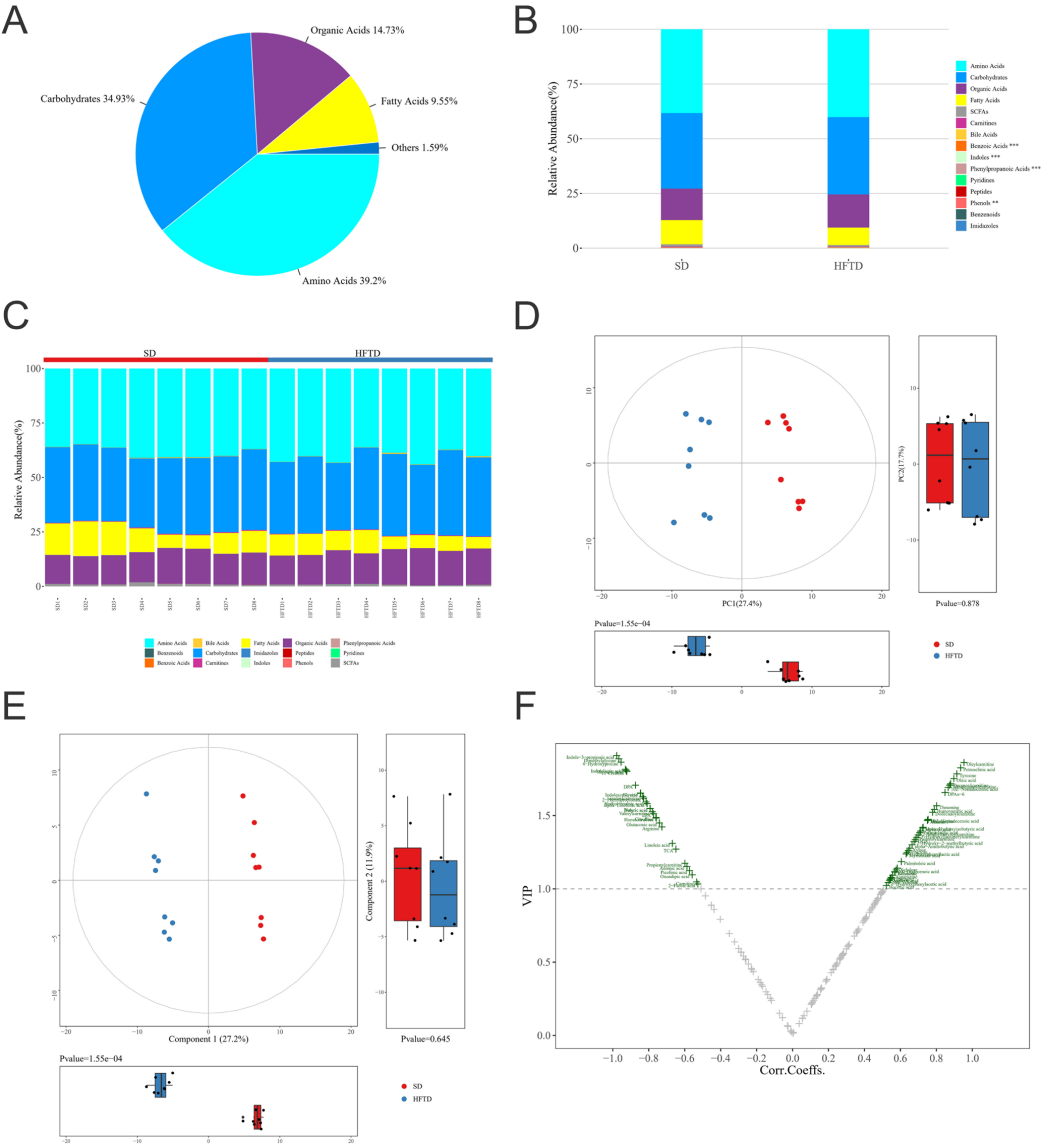
Metabolic profile analysis. (A) Pie chart of the mean abundance composition ratio of various metabolites in all samples. (B) Statistical stacked histograms of the median abundance of each type of metabolite in each group of samples. (C) Stacked histogram of the relative abundance of each metabolite type in each sample. (D) 2D principal component score plots of the analysed samples and box plots corresponding to the principal component scores. (E) Partial least square-discriminant analysis (PLS-DA) score with boxplot with points. (F) Based on the results of the orthogonal partial least squares-discriminant analysis (OPLS-DA) model, the Volcano plot was used to screen for reliable metabolic markers.

A total of 182 metabolites were identified in this study, and the mean abundance composition of each metabolite type in all the samples is shown in Fig. 3A, which shows that amino acids, carbohydrates, organic acids and fatty acids are the major components of the metabolite types, with proportions of 39.2%, 34.93%, 14.73% and 9.55%, respectively. The relative abundance of median values and the relative abundance of each type of metabolite were analysed in each sample (Fig. 3B and C). The categories that differed in serum metabolites from the two groups of rats mainly included amino acids, carbohydrates and fatty acids, confirming the effect of high fructose status on the three major metabolites.

##### Multidimensional statistics are analysed

Principal component analysis (PCA) was used to evaluate the clustering characteristics of serum samples from the rat model group and normal group. According to the PCA score plot with QC samples, it was found that in the unsupervised model without grouping conditions, the samples from the two groups could be effectively separated from each other, with the first two principal components corresponding to 23.3% and 16.1% of sample interpretation, while the QC sample points were close to each other and highly aggregated, suggesting good stability of the instrumental detection. Therefore, PCA was used to present 2D principal component score plots for the two groups of rat serum samples, with the two principal components corresponding to sample interpretation rates of 27.4% and 17.7%, respectively. The scores of all samples in each group were plotted further in the corresponding principal components as a box plot (Fig. 3D), which facilitated us to visually observe the mean level and degree of variation of the data from the two groups of rat serum samples, while the degree of variation was statistically significant (*P* < 0.05) for the model rat group samples compared with the control rat samples.

The PCA-based analysis method is an unsupervised evaluation method that often does not show a clear trend of separation between groups, especially when testing samples with complex backgrounds, regions, diets and other influencing factors. These influencing factors can cause the dataset to contain many noise signals that are not related to the group. In this case, the supervised classification discriminant models PLS-DA (partial least squares) and OPLS-DA (orthogonal partial least squares) were used. PLS-DA analysis can more obviously show the metabolite differences and trends in different groups of rats. The data of serum samples from both groups of rats were processed to obtain PLS-DA score plots with sample labels (Fig. 3E), and the results showed that there was a clear trend of separation between the model group rats and the control group rats in the first principal component (PC1) and second principal component (PC2) dimensions. Another supervised evaluation method, OPLS-DA, was further modified on the basis of PLS-DA, which was capable of filtering noise signals unrelated to the grouping, was suitable for modelling and differential metabolite screening between two groups, was easier to interpret and was a rigorous validation of the model. Therefore, the OPLS-DA model for the HFTD and the control group samples was established, and the differential metabolites between the two groups of rat samples were screened with the screening criteria of variable importance for the projection (VIP) > 1 and *P*-value < 0.05, and a volcano plot (Fig. 3F) was drawn for reliable metabolite marker screening. The volcano plots were combined to examine the contribution of metabolites to the model grouping (VIP) and the reliability of metabolites (correlation coefficients of metabolites with first principal component correlation), and metabolite VIP data results are shown in Supplementary Table 3. The model evaluation parameters R2Y and Q2 (R2Y indicates the explanation rate of the model for the Y variable, Q2 indicates the predictive ability of the model) obtained by the replacement test were 0.997 and 0.965, respectively, and both R2Y and Q2 were not less than 0.5, indicating that the model established by the study was stable and reliable (17, 18, 19, 20).

#### Screening for differential metabolites

##### Unidimensional statistical analysis

**Figure 4:**
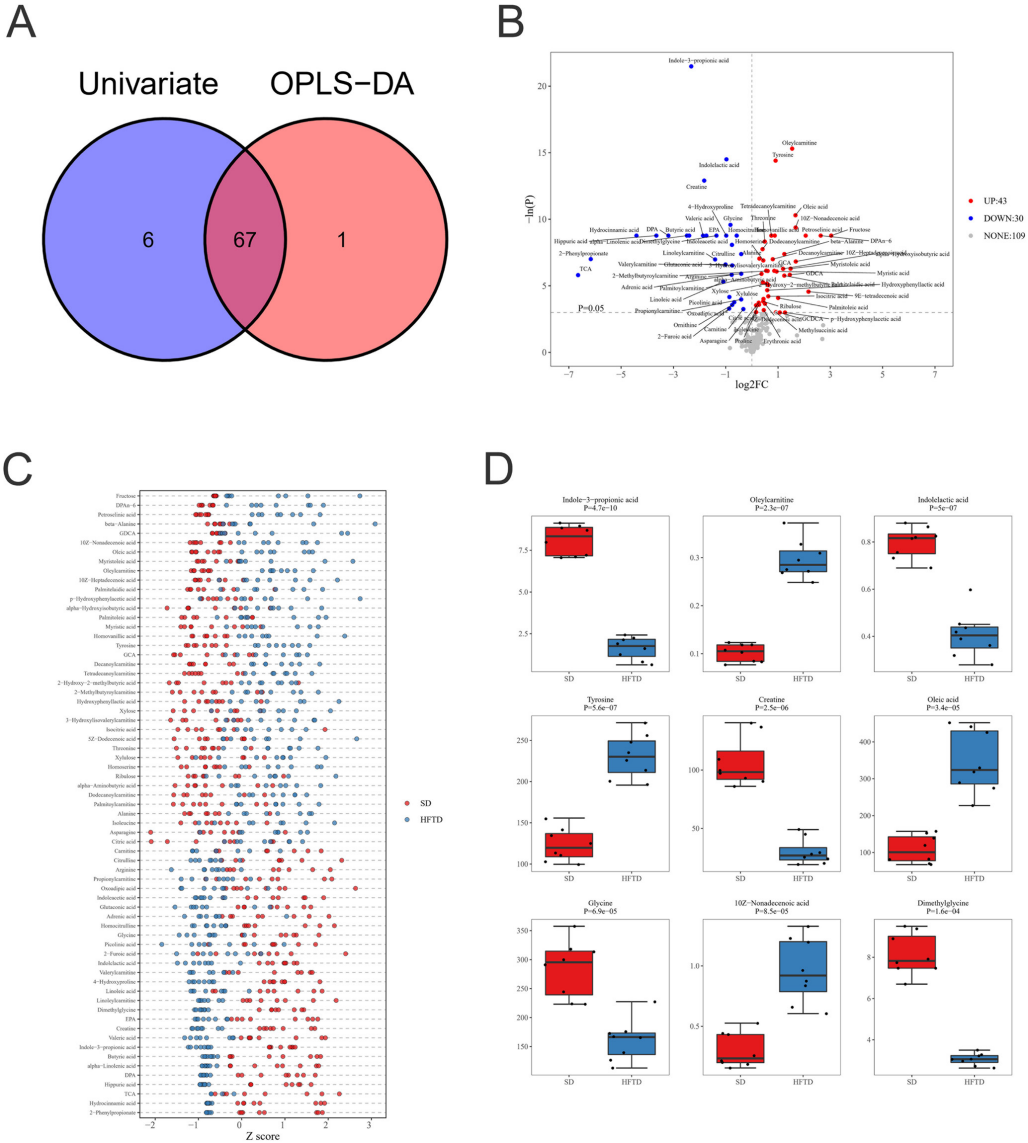
Differential metabolites and potential biomarkers. (A) Concatenated maps of differential metabolites acquired in one and multiple dimensions. (B) Metabolite volcano map with label. (C) Differential metabolite Z-score graph. (D) Box plots of the top nine differential metabolites for unidimensional statistical analysis of *P*-values.

Based on the multidimensional statistical analysis of serum samples from the two groups of rats, unidimensional statistical analysis was used for the screening of differential metabolites, and the two analysis methods were juxtaposed. From Fig. 4A, 67 differential metabolite intersections existed between the single and multidimensional analyses. On the other hand, the unidimensional test was used to obtain the differential metabolites between the two groups of rat samples for the 182 metabolites detected, and a volcano plot was built (Fig. 4B) to visualize the changes in metabolites in the two groups of rat samples. For this unidimensional analysis, the thresholds in the volcano plot graph were set as follows: (i) *P* < 0.05 and (ii) absolute value of log2FC > 0 (FC, fold change, i.e., between-group variation multiplier). In the figure, compared to the SD group, metabolites highlighted in the upper right corner are elevated in the HFTD group, and the metabolites highlighted in the upper left corner are decreased in the HFTD group. According to the screening criteria, a total of 73 differential metabolites were obtained from the unidimensional analysis of this study, and the *P*-values, basic information, and FC in the differential metabolites are shown in Supplementary Table 4. As seen in the volcano plot, compared to the control rats, 43 of the detected metabolites were upregulated, 30 metabolites were downregulated, all with *P*-values <0.05, and another 109 metabolites were not significantly different between the two groups. Since the screened differential metabolite data satisfied the normal distribution characteristics, the *Z* score point plots of the differential metabolites and the box plots of the top nine differential metabolites were plotted further (Fig. 4C and D). These nine metabolites were indole-2-propionic acid, carnitine oleate, indolinic acid, tyrosine, creatine, oleic acid, glycine, 10Z-nonadecanoic acid and dimethylglycine. The differential metabolites were mainly amino acids, fatty acids and organic acids.

##### Potential biomarkers

Differentiated metabolites were obtained using the intersection of the above unidimensional and multidimensional data, which was used because potential biomarkers may have biological significance on the basis of unidimensional and multidimensional analyses. The intersection of multidimensional and unidimensional criteria satisfies the screening of dual criteria and provides the most plausible differential metabolite, which is more likely to become a potential biomarker. The screening criteria for potential biomarkers were as follows: (i) unidimensional analysis *P* < 0.05, |log2FC| > 0 and (ii) multidimensional analysis VIP > 1. We finally obtained 67 metabolite intersections of potential biomarkers that met the set unidimensional and multidimensional screening criteria after several steps of correlation/partial correlation analysis, pathway enrichment analysis, pathway analysis and diagnostic experiments, and the raw data are shown in Supplementary Table 5. The top four potential biological markers with the highest differences in expression levels were oleoylcarnitine, tyrosine, oleic acid and 10Z-nonadecenoic acid, and the five biological markers with the lowest differences were indole-3-propionic acid, indolinic acid, creatine, glycine and dimethylglycine (Fig. 4D).

#### Metabolic pathway analysis

**Figure 5:**
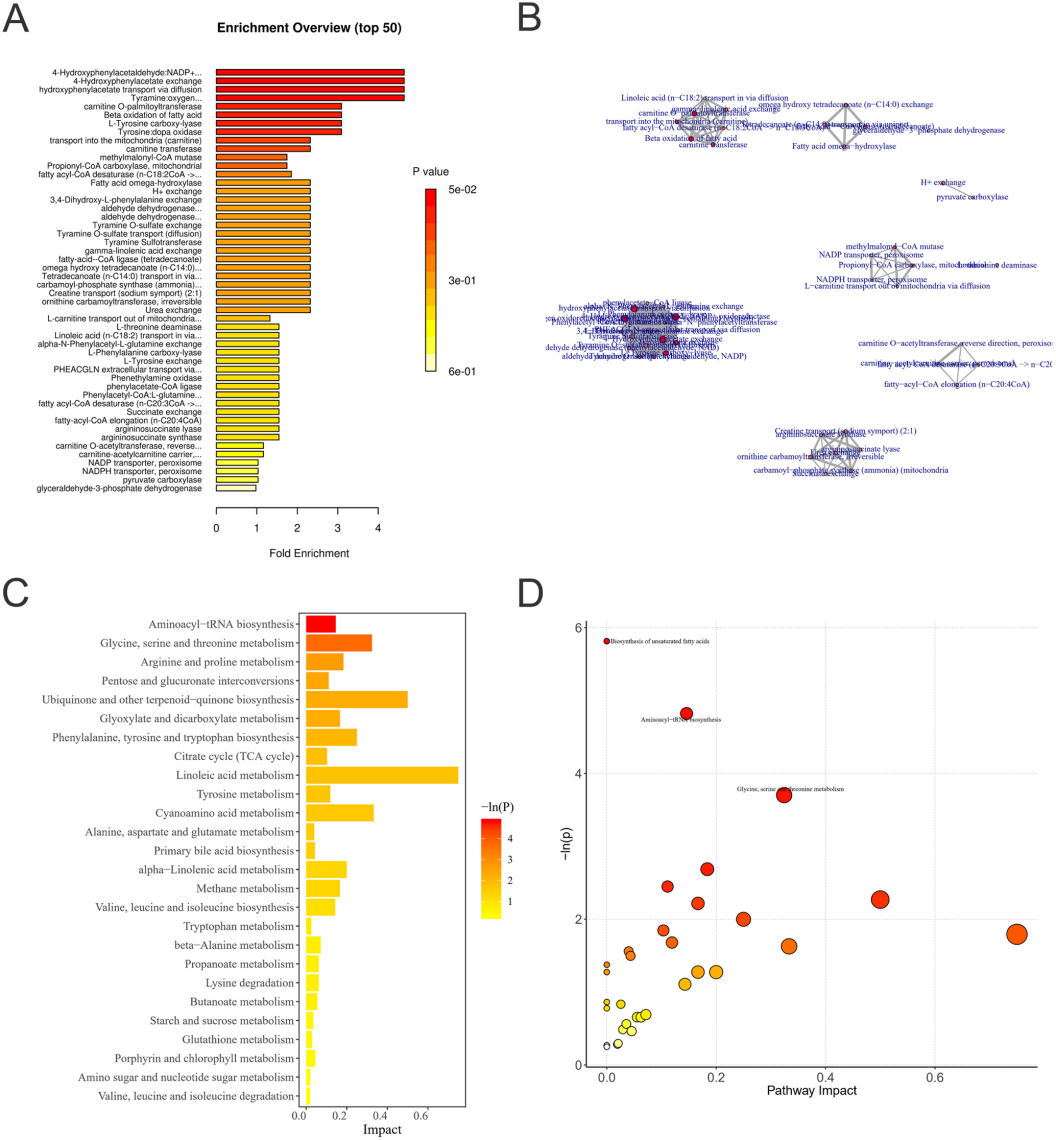
Metabolic pathway analysis. (A) Differential metabolite pathway enrichment analysis map. (B) Enrichment network diagram. (C) Passageway analysis bar chart. (D) Bubble diagram for pathway analysis.

Based on the results of the screened differential metabolites and potential biomarkers, the analysis of the relevant metabolic pathways was needed to further determine the possible mechanisms affecting metabolic disorders and elevated plasma BCAAs in rats fed a high-fructose diet in which the top 50 metabolites in terms of enrichment were listed (Fig. 5A). In order to visualize the pathway enrichment distribution, the metabolite pathway enrichment network was plotted (Fig. 5B), with the names of individual metabolites indicated in the figure. Then pathway analysis was performed by using the differential metabolites in the rno library, and the 26 metabolic pathways that met the screening criteria are listed in the bar graph (Fig. 5C). The metabolic pathways involved in the bubble graph were plotted (Fig. 5D), from which the three metabolic pathways with the most significant differences were finally found: namely, the glycine serine threonine metabolic pathway, the amino acid tRNA biosynthesis pathway and the unsaturated fatty acids pathway. The three most significantly different metabolic pathways were all involved in amino acid metabolism, which is closely related to insulin resistance, further supporting the metabolic phenotype of rats on a high-fructose diet and explaining the elevated plasma BCAA levels in rats.

### Discussion

In the present study, chronic consumption of a high-fructose diet led to a chronic increase in BCAA levels in the fasting plasma of high-fructose diet rats, which was mainly attributed to a decrease in the activity of a key enzyme for catabolism of BCAAs in rat skeletal muscle, as reported in a previous study (16). High-fructose diets are known to be a major cause of diabetes and various metabolic disorders, including fatty liver disease. However, except for elevated BCAA levels, the rats in the HFTD group showed weight gain and elevated total glyceride (TG), ALT, fasting blood glucose (FBG), RBG, insulin and HOMA-IR with time which are typical signs in metabolic diseases (21, 22, 23). The elevation of BCAAs occurs earlier than other metabolic disorders. Therefore, the rats fed a high-fructose diet for 8 weeks were ideal hyperbranched-chain amino acidaemia rat models used to investigate BCAAs and metabolic diseases. Based on the determination of model availability, four networks of enrichment pathways and nine potential biomarkers that may be involved in high BCAA levels affecting metabolic abnormalities were identified by applying metabolomics techniques.

Notably, this trend of chronically elevated BCAA levels during 8 weeks observed in the HFTD model, which was mainly attributed to a decrease in the activity of a key enzyme for catabolism of BCAA in rat skeletal muscle, was consistent with pathological changes in humans with metabolic diseases such as obesity and type 2 diabetes (24, 25). Consistent with our findings, recent studies have also shown that the catabolic pathway of BCAAs is defective in animal models of obesity and in the population, especially in adipose tissue, and is a cause of insulin resistance and diabetes mellitus (26). Moreover, the levels of TG, ALT, FBG, RBG, insulin and HOMA-IR in the HFTD group gradually increased and were higher than those in the control group at the end of the eighth week. However, BCAAs in the HFTD group gradually increased from the 4th week to the 8th week and were higher than those in the control group, which occurred earlier than other lipid and blood glucose changes. Therefore, it was inferred that these disorders of glycolipid metabolism were associated with elevated branched amino acids. The body weight of the rats in the HFTD group was significantly higher than that of the control group at the eighth week (*P* < 0.001). However, there was no significant difference between the two groups during the remaining weeks of feeding. Therefore, the hyperbranched-chain amino acidaemia rat model showed an obesity phenotype that was not evident (650 and 600 g) despite the presence of weight gain (<10%), and, therefore, it was inferred that the relationship between elevated circulating BCAA levels and disorders of glucolipid metabolism and insulin resistance was independent of obesity. In contrast, these metabolic disturbances associated with elevated BCAA levels were not observed in the PP2Cm^−/−^ mice (8, 15, 27), and the elevated level of BCAAs in the HFTD group was lower than that in PP2Cm^−/−^ mice (4, 15).

On the other hand, dietary supplementation with BCAAs is not an ideal way to construct an animal model of hyperbranched-chain amino acidaemia, because the effect of dietary BCAA intake on plasma BCAA levels has been found to be variable. In a study in which obese insulin-resistant rats were supplemented with double leucine, plasma leucine levels in the supplemented group were significantly higher than those in the unsupplemented group (28). This was unlike animal studies which found that even with BCAA supplementation, plasma BCAA levels were elevated only in the satiated state, with no significant difference between the supplemented and control groups during fasting (29). In a human trial involving 1997 female twins, daily BCAA intake was assessed using the Food Consumption Frequency Scale, plasma BCAA levels were measured, and no association was found between BCAA intake and circulating levels (30). The effect of BCAA supplementation on its plasma concentration is a matter of debate and may be related to different experimental designs, experimental animals, or population statuses. Therefore, based on what has been discussed above, the hyperbranched-chain amino acidaemia rat model constructed here was an ideal animal model to study the mechanisms of the association between BCAAs and metabolic diseases, as it was accompanied by progressively higher circulating BCAA levels and chronic metabolic disease characteristics consistent with the human population.

Elevated plasma BCAA levels are associated with the development of metabolic diseases, but the causal relationship and mechanism between BCAAs and cardiometabolic diseases are still not fully understood. In this study, the elevation of BCAAs occurred earlier than other metabolic disorders. To better reveal the possible mechanism, the abnormal metabolite alterations in rats with hyperbranched-chain amino acidaemia were analysed using metabolomics techniques. The Q300 metabolic microarray analysis revealed 73 metabolites that were differentially expressed in serum samples from the two groups of rats. The abnormal metabolite species in the HFTD group mainly included several compounds, such as carbohydrates, fatty acids, bile acids, amino acids, benzoic acid, carnosine and organic acids. Based on the differentially expressed metabolites, KEGG and GO functional enrichment analyses were performed to elucidate which specific signalling pathways may be involved in the pathogenesis of metabolic diseases regulated by high BCAA levels.

KEGG results showed that the differential metabolites were significantly enriched in pathways related to amino acids and bile acids, such as aminoacyl-tRNA biosynthesis, primary bile acid biosynthesis, arginine and proline metabolism and fatty acid biosynthesis. The aminoacyl-tRNA synthesis pathway is a prevalent cellular process that provides the substrate for ribosomal translation of mRNA during protein synthesis (31). Indeed, many studies have shown that this pathway can also be involved in various physiological and pathological processes, such as angiogenesis, inflammation, obesity, tumourigenesis, posttranslational modifications, translation initiation and autophagy regulation (32, 33, 34, 35, 36, 37). In addition, amyl-tRNA synthetase (ARS) is emerging as a multifunctional molecule involved in immune regulation and immune diseases (38, 39, 40). Notably, among ARSs, leucine tRNA synthetase 1 (LARS1) has been identified as a leucine sensor upstream of the amino acid sensing pathway and is thus involved in the coordinated control of protein synthesis and cell growth autophagy (41). In addition to LARS1, other types of ARSs may also be involved in intracellular homologous amino acid sensing and signalling. More importantly, ARSs have been reported to be closely associated with a variety of CVDs, which provides a theoretical basis for our clinical search on the mechanisms linking BCAA levels to the pathogenesis of CVDs through this pathway (42). Therefore, the aminoacyl-tRNA synthesis pathway of aberrant metabolite enrichment might be involved in the link between high BCAA levels and metabolic disorders.

As complex metabolic integrators and signal transducers, bile acids play an important role in many aspects of the intestine, including promoting lipid absorption, maintaining cholesterol homeostasis, inducing bile flow, excreting toxic substances and regulating energy metabolism (43). Recent advances in basic research have identified bile acids as nutrient sensors and metabolic integrators that activate farnesoid X receptor (FXR) and Takeda G protein-coupled receptor 5 (TGR5) to regulate lipid, glucose and energy metabolism and maintain metabolic homeostasis (44). Other studies have found that disorders of bile acid metabolism can lead to cholestatic liver disease, dyslipidaemia, fatty liver, CVD and diabetes (45). In this study, differential metabolites were significantly enriched in primary bile acid biosynthesis, suggesting that the bile acid signalling pathway may be involved in the mechanism of BCAA regulation of glucolipid metabolism and insulin sensitivity.

The arginine and proline metabolic pathway is another important protein metabolic pathway that may have implications for the development of obesity. l-proline plays an important role in energy regulation through its conversion to pyruvate. One signalling pathway through which l-arginine exerts beneficial metabolic effects may be the l-arginine–nitrogen oxide pathway. Through this pathway, l-arginine activates intracellular signalling proteins, restores insulin sensitivity, regulates glucose homeostasis, promotes lipolysis and maintains hormone levels (46), thus promoting a fat loss. In addition, a high-fat diet can reduce proline levels. Proline may also be a potential metabolite for obesity. The results of a study showed that urinary proline levels were reduced in a high-fat diet group of rats and that the FC of proline increased in a dose-dependent manner (47). Therefore, the arginine–proline metabolic pathway is also one of the pathways to explore in the future as a way for BCAAs to regulate organismal metabolism.

A final important differential metabolite pathway enrichment network is the biosynthesis of fatty acids, a major source of energy and an important component of membrane lipids, which also play an important role in the aetiology of metabolic syndrome as cellular signalling molecules. The fact that the accumulation of fat in tissues, such as muscle and liver, is associated with insulin resistance throughout the whole animal is widely accepted (48). However, recent evidence suggests that fatty acid synthesis plays an equally critical role in the function and survival of nonenergy-storing tissues (49). Acylation of proteins regulates their activation, localization and propensity for receptor binding (50). The HFTD rat model in this study exhibited typical dyslipidaemia, which on the one hand was related to long-term carbohydrate intake, such as fructose intake, and on the other hand revealed the involvement of fatty acid synthesis pathways in the regulatory network between BCAA and metabolic abnormalities, a mechanism that deserves to be explored in depth.

Based on the pathway enrichment analysis, the potential role of differential metabolites in rat serum was explored in diagnosing the association between BCAA levels and metabolic diseases. We categorized and summarized potential biomarkers and screened nine metabolites with the most significant differences, among which the metabolite levels were upregulated for oleoylcarnitine, tyrosine, oleic acid and 10Z-nonadecenoic acid and downregulated for indole-3-propionic acid, indolinic acid, creatine, glycine and dimethylglycine compared to the control rat samples. 3-Indolepropionic acid (IPA) or indole-3-propionic acid produced by the gut microbiota has been found to be a potent neuroprotective antioxidant, a plant growth hormone and a natural product in humans for the treatment of Alzheimer’s disease (51, 52, 53). As early as 2017, elevated plasma concentrations of IPA in humans were reported to be associated with a reduced risk of type 2 diabetes and an increased intake of fibre-rich foods (54, 55). Moreover, it has also been shown that the intestinal metabolite indole-3-propionic acid was identified as a mitochondrial regulator of cardiomyocytes that can have a direct effect on the physiology of the heart and alter cardiac function in an* in vivo* mouse model (56). Therefore, it has the potential to be one of the biomarkers used to assess the progression of metabolic diseases, such as CVDs. Glycine monitoring is becoming increasingly important as a biomarker in clinical analysis because of its involvement in multiple physiological functions, which makes glycine one of the most analysed diagnostic biomolecules. In a study on the amount of fat at different sites in elderly obese patients, it was confirmed that serum glycine can be used as a serum biomarker of insulin sensitivity and local fat mass (57). In conclusion, as an amino acid, glycine appears to have potential as a biomarker for the assessment of metabolic disorders associated with BCAAs.

In addition to the potential biomarkers mentioned above that were downregulated, some differential metabolite levels were upregulated in serum samples from rats with hyperbranched-chain amino acidaemia. Oleic acid is a monounsaturated long-chain fatty acid that has anti-inflammatory and preventive effects against insulin resistance and atherosclerosis, but the exact molecular mechanism of action is not known. In one study, oleic acid was found to specifically increase the rate of fatty acid oxidation in a sirt1 – pgc1 alpha-dependent manner (58). This may explain, at least in part, the protective effect of oleic acid against inflammation, dyslipidaemia and insulin resistance. Carnitine is essential for β-oxidative transport of long-chain fatty acids through the inner mitochondrial membrane. As one of the key players in fatty acid metabolism, oleoylcarnitine levels can not only distinguish patients with dilated cardiomyopathy from those with ischaemic cardiomyopathy but are also independent predictors of long-term mortality risk in patients with heart failure. Therefore, oleoylcarnitine may be a good early biomarker for cardiovascular metabolic diseases. Since metabolic diseases are usually present for many years before they become clinically apparent, new biomarkers of metabolic complications should be identified and labelled before the onset of metabolic diseases to facilitate early and effective interventions. The potential biomarkers identified in this study were statistically significant but not necessarily biochemically significant. Therefore, the clinical application of the screened metabolites requires more in-depth studies in the future and the inclusion of validation sets to identify more regulatory mechanisms between BCAAs and metabolic diseases.

This study also had some limitations. First, all experimental rats were male, and in the actual clinical population, females also represent a large proportion of metabolic diseases, such as obesity, diabetes and CVD. Second, since our goal was to model the association between high BCAA levels and metabolic diseases, our metabolomics technique was chosen for all-targeted serum analysis, which detected a small number of metabolite species and could not cover the whole metabolic pathway, and more compounds need to be detected and validated both *in vivo* and* in vitro* with relevant experiments. Therefore, based on the HFTD rats, we may later consider gender separation, expanding the types of metabolites tested and choosing a specific pathway enrichment network to dig deeper into the association between high BCAA levels and metabolic disorders.

### Conclusions

In conclusion, using a long-term high fructose diet, a hyperbranched-chain amino acidaemia rat model with multiple cardiovascular metabolic disease risk factors was successfully constructed in a nonobese state, which mimics the characteristics of a clinical disease population and is a reliable and stable animal model of metabolic disease. Based on the determination of model availability, four network enrichment pathways (aminoacyl-tRNA biosynthesis, primary bile acid biosynthesis, arginine and proline metabolism and fatty acid biosynthesis) and nine potential biomarkers that might be involved in high BCAA levels affecting metabolic abnormalities were identified by applying metabolomics techniques, which provided a theoretical basis for our in-depth mechanistic studies at a later stage.

### Supplementary Materials

Supplementary Table 1. The main composition of the standard diet and HFTD diet

Supplementary Table 2. Quantitative PCR primer sequences

Supplementary Table 3. VIP results data for metabolites of OPLS-DA

Supplementary Table 4. Specific information on 73 differential metabolites screened by unidimensional statistical analysis

Supplementary Table 5. Information on 67 potential markers obtained after unidimensional and multidimensional statistical analysis of the intersection

### Declaration of interest

The authors declare that the research was conducted in the absence of any commercial or financial relationships that could be construed as a potential conflict of interest.

### Funding

Research reported in this publication was supported by the Development Foundation Project of the Affiliated Hospital of Xuzhou Medical University (XYFM2021049), the Medical Research Project of Jiangsu Provincial Health and Family Planning Commission (H2018054) and effects of PPM1K rs1440581 and rs7678928 on serum branched-chain amino acid levels and risk of CVD (HAWJ202110).

### Data availability statement

The data analysed in this study are subject to the following licenses/restrictions: the data that support the findings of this study (all of the individual participant data collected during the study – after deidentification, statistical analysis plan and analytic code) are available from the corresponding author immediately following publication with the researchers who provide a methodologically sound proposal. Requests to access these datasets should be directed to Wen Hu, e-mail: huwen787878@163.com.

### Ethics statement

The study involving rats was reviewed and approved by the Animal Ethics Review Committee of Xuzhou Medical University.

### Publisher’s note

All claims expressed in this article are solely those of the authors and do not necessarily represent those of their affiliated organizations or those of the publisher, the editors and the reviewers. Any product that may be evaluated in this article, or claim that may be made by its manufacturer, is not guaranteed or endorsed by the publisher.

### Author contribution statement

Yang Yu wrote the manuscript. Wen Hu proofread the manuscript and provided guidance on the overall direction of the manuscript. All authors critically appraised the final version of the paper and approved the submitted version.
