# Supplementary material for: A metabolomics-based analysis of the metabolic pathways associated with the regulation of branched-chain amino acids in rats fed a high-fructose diet
Source: Endocr Connect. 2023 Sep 8;12(10):e230079. doi: 10.1530/EC-23-0079 (PMC10503218; doi:10.1530/EC-23-0079)
Supplement: Supplementary Table 1. The main composition of the standard diet and HFTD diet [file supplementary_table_1.pdf]

Supplementary Table 1. The main composition of the standard diet and HFTD diet

	Standard diet (%)	HFTD (%)
**Protein**	19	18.3
**Fat**	4	5.2
**Carbohydrate**	50	60.4
**Fiber**	5	3.2
**Ash content**	8	4.6
**Water content**	<10.0	3.2
**Calories per gram**	3.12kcal	3.6 kcal

Note: SD, standard diet; HFTD, high fructose diet.
