# Supplementary material for: A metabolomics-based analysis of the metabolic pathways associated with the regulation of branched-chain amino acids in rats fed a high-fructose diet
Source: Endocr Connect. 2023 Sep 8;12(10):e230079. doi: 10.1530/EC-23-0079 (PMC10503218; doi:10.1530/EC-23-0079)
Supplement: Supplementary Table 2. Quantitative PCR primer sequences [file supplementary_table_2.pdf]

Supplementary Table 2. Quantitative PCR primer sequences

Gene Symbol	Forward	Reverse
β-actin	TATAAAACCCGGCGGCGCA	TCATCCATGGCGAACTGGTG
ACAD	TGGAAGCCACACGGTTGCTAAC	CATCCACTCGATGCACTTGCTTG
BCKDH	ATGGCTATGCCATCTCCACACC	CAAACACATCGTTGCCGTCCAC
