# Supplementary material for: A metabolomics-based analysis of the metabolic pathways associated with the regulation of branched-chain amino acids in rats fed a high-fructose diet
Source: Endocr Connect. 2023 Sep 8;12(10):e230079. doi: 10.1530/EC-23-0079 (PMC10503218; doi:10.1530/EC-23-0079)
Supplement: Supplementary Table 3. VIP results data for metabolites of OPLS-DA [file supplementary_table_3.pdf]

Supplementary Table 3. VIP results data for metabolites of OPLS-DA

Class	Metabolite	HMDB	VIP	Corr.Coeffs.	P	FDR
Indoles	Indole-3-propionic acid	HMDB0002302	1.90	-0.98	5.90E-11	1.07E-08
Amino Acids	Dimethylglycine	HMDB0000092	1.88	-0.97	1.14E-09	1.04E-07
Amino Acids	4-Hydroxyproline	HMDB0000725	1.85	-0.95	9.19E-09	4.98E-07
Carnitines	Oleylcarnitine	HMDB0005065	1.85	0.95	1.09E-08	4.98E-07
Fatty Acids	Petroselinic acid	HMDB0002080	1.81	0.93	1.11E-07	4.03E-06
Indoles	Indolelactic acid	HMDB0000671	1.80	-0.93	1.82E-07	5.53E-06
Benzoic Acids	Hippuric acid	HMDB0000714	1.80	-0.93	2.69E-07	7.01E-06
Amino Acids	Creatine	HMDB0000064	1.79	-0.92	3.74E-07	8.50E-06
Amino Acids	Tyrosine	HMDB0000158	1.77	0.91	7.54E-07	1.52E-05
Fatty Acids	Oleic acid	HMDB0000207	1.74	0.90	2.49E-06	4.52E-05
Carnitines	Decanoylcarnitine	HMDB0000651	1.71	0.88	7.28E-06	1.20E-04
Carnitines	Tetradecanoylcarnitine	HMDB0005066	1.70	0.87	9.51E-06	1.35E-04
Fatty Acids	DPA	HMDB0006528	1.70	-0.87	9.66E-06	1.35E-04
Fatty Acids	10Z-Nonadecenoic acid	HMDB0013622	1.68	0.87	1.47E-05	1.91E-04
Fatty Acids	DPAn-6	HMDB0001976	1.65	0.85	3.30E-05	3.93E-04
Amino Acids	Glycine	HMDB0000123	1.64	-0.85	3.59E-05	3.93E-04
Indoles	Indoleacetic acid	HMDB0000197	1.64	-0.85	3.67E-05	3.93E-04
Carnitines	Linoleylcarnitine	HMDB0006469	1.62	-0.83	6.04E-05	6.11E-04
Phenylpropanoic Acids	2-Phenylpropionate	HMDB0011743	1.61	-0.83	7.33E-05	7.03E-04
Phenylpropanoic Acids	Hydrocinnamic acid	HMDB0000764	1.58	-0.82	1.19E-04	1.09E-03
Fatty Acids	alpha-Linolenic acid	HMDB0001388	1.57	-0.81	1.47E-04	1.27E-03
Amino Acids	Threonine	HMDB0000167	1.56	0.80	1.89E-04	1.56E-03
SCFAs	Butyric acid	HMDB0000039	1.54	-0.79	2.53E-04	1.94E-03
SCFAs	Valeric acid	HMDB0000892	1.54	-0.79	2.56E-04	1.94E-03
Phenols	Homovanillic acid	HMDB0000118	1.53	0.79	2.83E-04	2.06E-03
Carnitines	Valerylcarnitine	HMDB0013128	1.51	-0.78	3.63E-04	2.53E-03
Carnitines	Dodecanoylcarnitine	HMDB0002250	1.51	0.78	3.76E-04	2.53E-03
Fatty Acids	EPA	HMDB0001999	1.50	-0.77	4.37E-04	2.84E-03
Amino Acids	Citrulline	HMDB0000904	1.48	-0.76	6.30E-04	3.95E-03
Amino Acids	Homocitrulline	HMDB0000679	1.47	-0.76	6.61E-04	4.01E-03
Fatty Acids	10Z-Heptadecenoic acid	HMDB0060038	1.46	0.75	7.45E-04	4.35E-03
Amino Acids	Homoserine	HMDB0000719	1.46	0.75	7.64E-04	4.35E-03
Amino Acids	Alanine	HMDB0000161	1.46	0.75	8.19E-04	4.51E-03
Organic Acids	Glutaconic acid	HMDB0000620	1.44	-0.74	1.02E-03	5.43E-03
Amino Acids	Arginine	HMDB0000517	1.41	-0.73	1.42E-03	7.34E-03
Organic Acids	alpha-Hydroxyisobutyric acid	HMDB0000729	1.41	0.73	1.45E-03	7.34E-03
Fatty Acids	Myristic acid	HMDB0000806	1.40	0.72	1.53E-03	7.52E-03
Fatty Acids	Palmitelaidic acid	HMDB0012328	1.38	0.71	1.93E-03	9.26E-03
Carnitines	2-Methylbutyroylcarnitine	HMDB0000378	1.37	0.71	2.18E-03	1.02E-02
Carnitines	3-Hydroxylisovalerylcarnitine	NA	1.36	0.70	2.60E-03	1.18E-02
Carnitines	Palmitoylcarnitine	HMDB0000222	1.34	0.69	3.07E-03	1.36E-02
Bile Acids	GCA	HMDB0000138	1.32	0.68	3.58E-03	1.55E-02
Organic Acids	2-Hydroxy-2-methylbutyric acid	HMDB0001987	1.31	0.68	4.05E-03	1.72E-02

Fatty Acids	Linoleic acid	HMDB0000673	1.30	-0.67	4.58E-03	1.90E-02
Amino Acids	alpha-Aminobutyric acid	HMDB0000452	1.29	0.66	4.96E-03	2.01E-02
Carbohydrates	Xylose	HMDB0000098	1.27	0.65	6.19E-03	2.45E-02
Bile Acids	TCA	HMDB0000036	1.26	-0.65	6.49E-03	2.51E-02
Carbohydrates	Fructose	HMDB0000660	1.25	0.64	7.07E-03	2.68E-02
Phenylpropanoic Acids	Hydroxyphenyllactic acid	HMDB0000755	1.24	0.64	7.95E-03	2.95E-02
Fatty Acids	Myristoleic acid	HMDB0002000	1.23	0.63	8.58E-03	3.12E-02
Fatty Acids	Palmitoleic acid	HMDB0003229	1.17	0.60	1.30E-02	4.65E-02
Carnitines	Propionylcarnitine	HMDB0000824	1.16	-0.60	1.41E-02	4.94E-02
Fatty Acids	Adrenic acid	HMDB0002226	1.14	-0.59	1.66E-02	5.69E-02
Carbohydrates	Xylulose	HMDB0001644	1.13	0.58	1.79E-02	6.03E-02
Fatty Acids	5Z-Dodecenoic acid	HMDB0000529	1.12	0.58	1.95E-02	6.46E-02
Pyridines	Picolinic acid	HMDB0002243	1.11	-0.57	2.02E-02	6.56E-02
Carbohydrates	Ribulose	HMDB0000621	1.11	0.57	2.12E-02	6.76E-02
Amino Acids	Isoleucine	HMDB0000172	1.10	0.57	2.15E-02	6.76E-02
Organic Acids	Oxoadipic acid	HMDB0000225	1.08	-0.56	2.44E-02	7.53E-02
Amino Acids	Asparagine	HMDB0000168	1.08	0.56	2.49E-02	7.55E-02
Organic Acids	Citric acid	HMDB0000094	1.07	0.55	2.76E-02	8.22E-02
Bile Acids	GDCA	HMDB0000631	1.06	0.55	2.80E-02	8.22E-02
Amino Acids	Aspartic acid	HMDB0000191	1.06	0.54	2.95E-02	8.52E-02
Amino Acids	beta-Alanine	HMDB0000056	1.05	0.54	3.09E-02	8.79E-02
Carnitines	Carnitine	HMDB0000062	1.04	-0.53	3.32E-02	9.28E-02
Phenols	p-Hydroxyphenylacetic acid	HMDB0000020	1.03	0.53	3.39E-02	9.35E-02
Organic Acids	2-Furoic acid	HMDB0000617	1.02	-0.53	3.61E-02	9.79E-02
Organic Acids	Isocitric acid	HMDB0000193	1.01	0.52	3.83E-02	1.02E-01
Amino Acids	Ornithine	HMDB0000214	0.99	-0.51	4.39E-02	1.16E-01
Carbohydrates	Erythronic acid	HMDB0000613	0.98	0.51	4.57E-02	1.19E-01
Fatty Acids	9E-tetradecenoic acid	HMDB0062248	0.97	0.50	4.81E-02	1.23E-01
Organic Acids	Ketoleucine	HMDB0000695	0.97	0.50	4.87E-02	1.23E-01
Fatty Acids	Methylsuccinic acid	HMDB0001844	0.97	0.50	4.96E-02	1.23E-01
Bile Acids	HDCA	HMDB0000733	0.97	0.50	4.98E-02	1.23E-01
