# Supplementary material for: A metabolomics-based analysis of the metabolic pathways associated with the regulation of branched-chain amino acids in rats fed a high-fructose diet
Source: Endocr Connect. 2023 Sep 8;12(10):e230079. doi: 10.1530/EC-23-0079 (PMC10503218; doi:10.1530/EC-23-0079)
Supplement: Supplementary Table 4. Specific information on 73 differential metabolites screened by unidimensional statistical analysis [file supplementary_table_4.pdf]

Supplementary Table 4. Specific information on 73 differential metabolites screened by unidimensional statistical analysis

Class	Metabolite	P	FDR	FC	log2FC	test.method
Indoles	Indole-3-propionic acid	4.66E-10	8.48E-08	0.2	-2.32	t.test
Carnitines	Oleylcarnitine	2.25E-07	2.05E-05	2.91	1.54	t.test
Indoles	Indolelactic acid	5.03E-07	2.53E-05	0.51	-0.97	t.test
Amino Acids	Tyrosine	5.55E-07	2.53E-05	1.87	0.91	t.test
Amino Acids	Creatine	2.49E-06	9.07E-05	0.28	-1.82	t.test
Fatty Acids	Oleic acid	3.37E-05	1.02E-03	3.17	1.67	t.test
Amino Acids	Glycine	6.91E-05	1.23E-03	0.57	-0.82	t.test
Fatty Acids	10Z-Nonadecenoic acid	8.47E-05	1.23E-03	3.19	1.67	t.test
Amino Acids	Dimethylglycine	1.55E-04	1.23E-03	0.39	-1.35	wilcox.test
Amino Acids	Homocitrulline	1.55E-04	1.23E-03	0.67	-0.58	wilcox.test
Benzoic Acids	Hippuric acid	1.55E-04	1.23E-03	0.08	-3.65	wilcox.test
Phenylpropanoic Acids	Hydrocinnamic acid	1.55E-04	1.23E-03	0.05	-4.41	wilcox.test
Carbohydrates	Fructose	1.55E-04	1.23E-03	8.18	3.03	wilcox.test
Phenols	Homovanillic acid	1.55E-04	1.23E-03	1.83	0.87	wilcox.test
SCFAs	Butyric acid	1.55E-04	1.23E-03	0.19	-2.4	wilcox.test
SCFAs	Valeric acid	1.55E-04	1.23E-03	0.27	-1.87	wilcox.test
Fatty Acids	alpha-Linolenic acid	1.55E-04	1.23E-03	0.18	-2.49	wilcox.test
Fatty Acids	EPA	1.55E-04	1.23E-03	0.3	-1.74	wilcox.test
Fatty Acids	DPA	1.55E-04	1.23E-03	0.11	-3.19	wilcox.test
Fatty Acids	DPAn-6	1.55E-04	1.23E-03	6.2	2.63	wilcox.test
Fatty Acids	Petroselinic acid	1.55E-04	1.23E-03	4.16	2.06	wilcox.test
Carnitines	Tetradecanoylcarnitine	1.55E-04	1.23E-03	1.68	0.75	wilcox.test
Amino Acids	4-Hydroxyproline	1.55E-04	1.23E-03	0.51	-0.98	wilcox.test
Amino Acids	Threonine	2.43E-04	1.84E-03	1.41	0.49	t.test
Indoles	Indoleacetic acid	3.11E-04	2.26E-03	0.59	-0.76	wilcox.test
Carnitines	Dodecanoylcarnitine	4.29E-04	3.01E-03	1.33	0.41	t.test
Amino Acids	beta-Alanine	6.22E-04	4.04E-03	2.36	1.24	wilcox.test
Amino Acids	Citrulline	6.22E-04	4.04E-03	0.75	-0.41	wilcox.test
Amino Acids	Alanine	8.75E-04	5.29E-03	1.22	0.29	t.test
Phenylpropanoic Acids	2-Phenylpropionate	9.07E-04	5.29E-03	0.01	-6.16	wilcox.test
Carnitines	Decanoylcarnitine	9.15E-04	5.29E-03	1.74	0.8	wilcox.test
Carnitines	Linoleylcarnitine	9.31E-04	5.29E-03	0.38	-1.41	wilcox.test
Amino Acids	Homoserine	1.00E-03	5.53E-03	1.35	0.44	t.test
Fatty Acids	10Z-Heptadecenoic acid	1.09E-03	5.82E-03	3.2	1.68	wilcox.test
Carnitines	Valerylcarnitine	1.36E-03	7.07E-03	0.5	-1.01	t.test
Organic Acids	Glutaconic acid	1.46E-03	7.39E-03	0.59	-0.75	t.test
Fatty Acids	Myristoleic acid	1.86E-03	9.02E-03	2.79	1.48	wilcox.test
Organic Acids	alpha-Hydroxyisobutyric acid	1.88E-03	9.02E-03	2.29	1.19	t.test
Carnitines	3-Hydroxylisovalerylcarnitine	2.17E-03	9.83E-03	1.46	0.55	t.test
Bile Acids	GCA	2.19E-03	9.83E-03	1.81	0.85	t.test
Carnitines	2-Methylbutyroylcarnitine	2.21E-03	9.83E-03	1.52	0.61	t.test
Fatty Acids	Myristic acid	2.30E-03	9.96E-03	1.92	0.94	t.test
Amino Acids	Arginine	2.73E-03	1.13E-02	0.75	-0.41	t.test

Carnitines	Palmitoylcarnitine	2.74E-03	1.13E-02	1.29	0.37	t.test
Bile Acids	GDCA	2.95E-03	1.17E-02	2.73	1.45	wilcox.test
Fatty Acids	Adrenic acid	2.95E-03	1.17E-02	0.59	-0.77	wilcox.test
Bile Acids	TCA	3.02E-03	1.17E-02	0.01	-6.65	wilcox.test
Fatty Acids	Palmitelaidic acid	3.14E-03	1.19E-02	2.36	1.24	t.test
Fatty Acids	Linoleic acid	4.96E-03	1.84E-02	0.47	-1.09	t.test
Amino Acids	alpha-Aminobutyric acid	5.20E-03	1.89E-02	1.35	0.43	t.test
Carbohydrates	Xylose	5.75E-03	2.05E-02	1.49	0.58	t.test
Organic Acids	2-Hydroxy-2-methylbutyric acid	6.33E-03	2.22E-02	1.54	0.63	t.test
Phenylpropanoic Acids	Hydroxyphenyllactic acid	9.34E-03	3.21E-02	1.5	0.59	t.test
Fatty Acids	9E-tetradecenoic acid	1.04E-02	3.51E-02	4.48	2.16	wilcox.test
Organic Acids	Isocitric acid	1.48E-02	4.89E-02	1.55	0.63	wilcox.test
Pyridines	Picolinic acid	1.56E-02	5.06E-02	0.55	-0.86	t.test
Fatty Acids	Palmitoleic acid	1.67E-02	5.33E-02	2	1	t.test
Carbohydrates	Xylulose	1.80E-02	5.65E-02	1.36	0.44	t.test
Carnitines	Propionylcarnitine	1.87E-02	5.76E-02	0.75	-0.41	t.test
Carbohydrates	Ribulose	2.18E-02	6.60E-02	1.35	0.43	t.test
Organic Acids	Oxoadipic acid	2.30E-02	6.87E-02	0.63	-0.67	t.test
Amino Acids	Asparagine	2.39E-02	7.02E-02	1.2	0.26	t.test
Fatty Acids	5Z-Dodecenoic acid	2.57E-02	7.44E-02	1.41	0.5	t.test
Amino Acids	Isoleucine	2.78E-02	7.88E-02	1.21	0.27	t.test
Amino Acids	Ornithine	2.81E-02	7.88E-02	0.59	-0.75	wilcox.test
Organic Acids	Citric acid	2.90E-02	8.01E-02	1.12	0.16	t.test
Organic Acids	2-Furoic acid	3.72E-02	1.01E-01	0.55	-0.87	t.test
Carnitines	Carnitine	3.87E-02	1.04E-01	0.8	-0.33	t.test
Carbohydrates	Erythronic acid	4.17E-02	1.10E-01	1.37	0.46	t.test
Amino Acids	Proline	4.84E-02	1.24E-01	1.12	0.16	t.test
Fatty Acids	Methylsuccinic acid	4.92E-02	1.24E-01	1.89	0.92	t.test
