# Supplementary material for: A metabolomics-based analysis of the metabolic pathways associated with the regulation of branched-chain amino acids in rats fed a high-fructose diet
Source: Endocr Connect. 2023 Sep 8;12(10):e230079. doi: 10.1530/EC-23-0079 (PMC10503218; doi:10.1530/EC-23-0079)
Supplement: Supplementary Table 5. Information on 67 potential markers obtained after unidimensional and multidimensional statistical analysis of the intersection [file supplementary_table_5.pdf]

Supplementary Table 5. Information on 67 potential markers obtained after unidimensional and multidimensional statistical analysis of the intersection

HMDB	KEGG	Metabolite	Uni_P	Uni_FDR	FC	log2FC
HMDB0002302	NA	Indole-3-propionic acid	4.66E-10	8.48E-08	0.20	-2.32
HMDB0005065	NA	Oleylcarnitine	2.25E-07	2.05E-05	2.91	1.54
HMDB0000671	C02043	Indolelactic acid	5.03E-07	2.53E-05	0.51	-0.97
HMDB0000158	C00082	Tyrosine	5.55E-07	2.53E-05	1.87	0.91
HMDB0000064	C00300	Creatine	2.49E-06	9.07E-05	0.28	-1.82
HMDB0000207	C00712	Oleic acid	3.37E-05	1.02E-03	3.17	1.67
HMDB0000123	C00037	Glycine	6.91E-05	1.23E-03	0.57	-0.82
HMDB0013622	NA	10Z-Nonadecenoic acid	8.47E-05	1.23E-03	3.19	1.67
HMDB0000092	C01026	Dimethylglycine	1.55E-04	1.23E-03	0.39	-1.35
HMDB0000679	C02427	Homocitrulline	1.55E-04	1.23E-03	0.67	-0.58
HMDB0000714	C01586	Hippuric acid	1.55E-04	1.23E-03	0.08	-3.65
HMDB0000764	C05629	Hydrocinnamic acid	1.55E-04	1.23E-03	0.05	-4.41
HMDB0000660	C02336	Fructose	1.55E-04	1.23E-03	8.18	3.03
HMDB0000118	C05582	Homovanillic acid	1.55E-04	1.23E-03	1.83	0.87
HMDB0000039	C00246	Butyric acid	1.55E-04	1.23E-03	0.19	-2.40
HMDB0000892	C00803	Valeric acid	1.55E-04	1.23E-03	0.27	-1.87
HMDB0001388	C06427	alpha-Linolenic acid	1.55E-04	1.23E-03	0.18	-2.49
HMDB0001999	C06428	EPA	1.55E-04	1.23E-03	0.30	-1.74
HMDB0006528	C16513	DPA	1.55E-04	1.23E-03	0.11	-3.19
HMDB0001976	NA	DPAn-6	1.55E-04	1.23E-03	6.20	2.63
HMDB0002080	C08363	Petroselinic acid	1.55E-04	1.23E-03	4.16	2.06
HMDB0005066	NA	Tetradecanoylcarnitine	1.55E-04	1.23E-03	1.68	0.75
HMDB0000725	C01157	4-Hydroxyproline	1.55E-04	1.23E-03	0.51	-0.98
HMDB0000167	C00188	Threonine	2.43E-04	1.84E-03	1.41	0.49
HMDB0000197	C00954	Indoleacetic acid	3.11E-04	2.26E-03	0.59	-0.76
HMDB0002250	NA	Dodecanoylcarnitine	4.29E-04	3.01E-03	1.33	0.41
HMDB0000056	C00099	beta-Alanine	6.22E-04	4.04E-03	2.36	1.24
HMDB0000904	C00327	Citrulline	6.22E-04	4.04E-03	0.75	-0.41
HMDB0000161	C00041	Alanine	8.75E-04	5.29E-03	1.22	0.29
HMDB0011743	NA	2-Phenylpropionate	9.07E-04	5.29E-03	0.01	-6.16
HMDB0000651	NA	Decanoylcarnitine	9.15E-04	5.29E-03	1.74	0.80
HMDB0006469	NA	Linoleylcarnitine	9.31E-04	5.29E-03	0.38	-1.41
HMDB0000719	C00263	Homoserine	1.00E-03	5.53E-03	1.35	0.44
HMDB0060038	NA	10Z-Heptadecenoic acid	1.09E-03	5.82E-03	3.20	1.68
HMDB0013128	NA	Valerylcarnitine	1.36E-03	7.07E-03	0.50	-1.01
HMDB0000620	C02214	Glutaconic acid	1.46E-03	7.39E-03	0.59	-0.75
HMDB0002000	C08322	Myristoleic acid	1.86E-03	9.02E-03	2.79	1.48
HMDB0000729	NA	alpha-Hydroxyisobutyric acid	1.88E-03	9.02E-03	2.29	1.19
NA	NA	3-Hydroxylisovalerylcarnitine	2.17E-03	9.83E-03	1.46	0.55
HMDB0000138	C01921	GCA	2.19E-03	9.83E-03	1.81	0.85
HMDB0000378	NA	2-Methylbutyroylcarnitine	2.21E-03	9.83E-03	1.52	0.61
HMDB0000806	C06424	Myristic acid	2.30E-03	9.96E-03	1.92	0.94

HMDB0000517	C00062	Arginine	2.73E-03	1.13E-02	0.75	-0.41
HMDB0000222	C02990	Palmitoylcarnitine	2.74E-03	1.13E-02	1.29	0.37
HMDB0000631	C05464	GDCA	2.95E-03	1.17E-02	2.73	1.45
HMDB0002226	C16527	Adrenic acid	2.95E-03	1.17E-02	0.59	-0.77
HMDB0000036	C05122	TCA	3.02E-03	1.17E-02	0.01	-6.65
HMDB0012328	NA	Palmitelaidic acid	3.14E-03	1.19E-02	2.36	1.24
HMDB0000673	C01595	Linoleic acid	4.96E-03	1.84E-02	0.47	-1.09
HMDB0000452	C02356	alpha-Aminobutyric acid	5.20E-03	1.89E-02	1.35	0.43
HMDB0000098	C00181	Xylose	5.75E-03	2.05E-02	1.49	0.58
HMDB0001987	NA	2-Hydroxy-2-methylbutyric acid	6.33E-03	2.22E-02	1.54	0.63
HMDB0000755	C03672	Hydroxyphenyllactic acid	9.34E-03	3.21E-02	1.50	0.59
HMDB0000193	C00311	Isocitric acid	1.48E-02	4.89E-02	1.55	0.63
HMDB0002243	C10164	Picolinic acid	1.56E-02	5.06E-02	0.55	-0.86
HMDB0003229	C08362	Palmitoleic acid	1.67E-02	5.33E-02	2.00	1.00
HMDB0001644	C00310	Xylulose	1.80E-02	5.65E-02	1.36	0.44
HMDB0000824	C03017	Propionylcarnitine	1.87E-02	5.76E-02	0.75	-0.41
HMDB0000621	C00309	Ribulose	2.18E-02	6.60E-02	1.35	0.43
HMDB0000225	C00322	Oxoadipic acid	2.30E-02	6.87E-02	0.63	-0.67
HMDB0000168	C00152	Asparagine	2.39E-02	7.02E-02	1.20	0.26
HMDB0000529	NA	5Z-Dodecenoic acid	2.57E-02	7.44E-02	1.41	0.50
HMDB0000172	C00407	Isoleucine	2.78E-02	7.88E-02	1.21	0.27
HMDB0000094	C00158	Citric acid	2.90E-02	8.01E-02	1.12	0.16
HMDB0000617	C01546	2-Furoic acid	3.72E-02	1.01E-01	0.55	-0.87
HMDB0000062	C00318	Carnitine	3.87E-02	1.04E-01	0.80	-0.33
HMDB0000020	C00642	p-Hydroxyphenylacetic acid	4.99E-02	1.24E-01	2.40	1.27
